# The contribution of TWIK-1 channels to astrocyte K^+^ current is limited by retention in intracellular compartments

**DOI:** 10.3389/fncel.2013.00246

**Published:** 2013-12-09

**Authors:** Wei Wang, Adhytia Putra, Gary P. Schools, Baofeng Ma, Haijun Chen, Leonard K. Kaczmarek, Jacques Barhanin, Florian Lesage, Min Zhou

**Affiliations:** ^1^Department of Neuroscience, The Ohio State University Wexner Medical CenterColumbus, OH, USA; ^2^South Carolina College of PharmacyColumbia, SC, USA; ^3^Department of Biological Sciences, University at Albany, SUNYAlbany, NY, USA; ^4^Department of Pharmacology, Yale University School of MedicineNew Haven, CT, USA; ^5^Centre National de la Recherche Scientifique, Institut de Pharmacologie Moléculaire et Cellulaire, Université de Nice Sophia AntipolisValbonne, France

**Keywords:** astrocytes, TWIK-1 potassium channel, patch clamp, western blot, qRT-PCR

## Abstract

TWIK-1 two-pore domain K^+^ channels are expressed abundantly in astrocytes. In the present study, we examined the extent to which TWIK-1 contributes to the linear current-voltage (I–V) relationship (passive) K^+^ membrane conductance, a dominant electrophysiological feature of mature hippocampal astrocytes. Astrocytes from TWIK-1 knockout mice have a more negative resting potential than those from wild type animals and a reduction in both inward rectification and Cs^+^ permeability. Nevertheless, the overall whole-cell passive conductance is not altered significantly in TWIK-1 knockout astrocytes. The expression of K_ir_4.1 and TREK-1, two other major astrocytic K^+^ channels, or of other two-pore K^+^ channels is not altered in TWIK-1 knockout mice, suggesting that the mild effect of TWIK-1 knockout does not result from compensation by these channels. Fractionation experiments showed that TWIK-1 is primarily localized in intracellular cytoplasmic fractions (55%) and mildly hydrophobic internal compartment fractions (41%), with only 5% in fractions containing plasma membranes. Our study revealed that TWIK-1 proteins are mainly located in the intracellular compartments of hippocampal astrocyte under physiological condition, therefore a minimal contribution of TWIK-1 channels to whole-cell currents is likely attributable to a relatively low level presence of channels in the plasma membrane.

## Introduction

Astrocytes are the most numerous glial subtype in the mammalian brain, where they play key roles in K^+^ and neurotransmitter homeostasis, synaptic transmission modulation, and neurovascular signaling (Perters et al., 1991; Haydon and Carmignoto, 2006; Wang and Bordey, 2008; Kimelberg, 2010). In rodent hippocampus, functional mature astrocytes are characterized by a highly negative resting membrane potential, low membrane resistance and a close to linear current-to-voltage (I–V) relationship (passive conductance) (Steinhauser et al., 1992; Zhou et al., 2006; Kafitz et al., 2008), indicating a high resting potassium conductance of the plasma membrane. Among the K^+^ channel subunits known to be expressed in astrocytes, inwardly rectifier K_ir_4.1, TWIK-1 and TREK-1 two-pore domain K^+^ channels (K_2P_) are the current focus of intense investigation (Connors et al., 2004; Skatchkov et al., 2006; Djukic et al., 2007; Cahoy et al., 2008; Seifert et al., 2009; Zhou et al., 2009; Chever et al., 2010; Chu et al., 2010; Woo et al., 2012; Wu et al., 2013). However, the molecular identity of the channels responsible for the passive conductance is not yet fully understood.

In mouse brain, TWIK-1 mRNA is the most abundantly expressed K^+^ channel mRNA in astrocytes (Cahoy et al., 2008). TWIK-1 exhibits several unusual biophysical features among K_2P_ channels. First, TWIK-1 behaves as a weak inward rectifying K^+^ channel in heterologous systems (Lesage et al., 1996; Rajan et al., 2005; Ma et al., 2011) while a typical K_2P_ channel follows the Goldman-Hodgkin-Katz (GHK) constant field rectification in physiological K^+^ solutions. Second, TWIK-1 produces only modest currents in oocytes and almost no current in mammalian cells (Lesage et al., 1996; Ma et al., 2011). Third, TWIK-1 channel switches to conduct Na^+^ ions in acidic or low K^+^ concentration bath solutions (Ma et al., 2011; Chatelain et al., 2012).

We have previously shown that TWIK-1 and TREK-1 proteins are expressed in rat hippocampal astrocytes. Additionally, the astrocyte passive conductance shares several similarities with that of cloned TWIK-1 and TREK-1 channels in heterologous expression systems, leading to the notion that these channels functionally contribute to the passive conductance (Zhou et al., 2009). However, a lack of specific blockers prevented a direct test of this hypothesis.

In this study we have taken the advantage of TWIK-1 knockout (TWIK-1^−/−^) mice to examine the contribution of this channel to the basic electrophysiological properties of mice hippocampal astrocytes *in situ*. We now show that, although TWIK-1 knockout produced perceptible changes in electrophysiological properties, the overall level of passive conductance is not altered remarkably. We further show that TWIK-1 proteins are mainly located in cytoplasmic fractions, indicating its retention in intracellular organelles. Therefore a relatively low level of expression in the plasma membrane is likely to underlie the relatively minor contribution of TWIK-1 to the electrophysiological properties of mature astrocytes *in situ*.

## Materials and methods

### Animals

**Figure 1:**
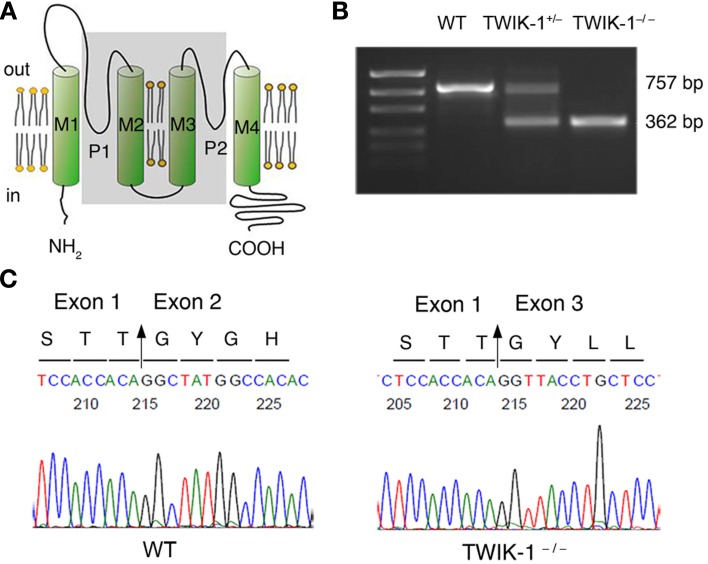
**TWIK-1 gene knockout mice. (A)** Schematic illustration of the targeted exon 2 deletion in TWIK-1 gene. Exon 2 encodes the two ion conducting pores (P1, P2), and the transmembrane domain 2 and 3 (M2, M3) (all in shadow). **(B)** RT-PCR amplification targeted on the axon 2 of TWIK-1 gene was performed with the mRNAs extracted from hippocampus of WT, heterozygote (TWIK-1^+/−^) and TWIK-1^−/−^ mice, that yielded the anticipated products as indicated. **(C)** Sequencing analysis from the PCR products from **(B)** confirmed an anticipated deletion of exon 2 in TWIK-1^−/−^ mouse, which encodes 132 amino acids in the position 119–250 of TWIK-1 channel.

The animal experiments were performed in accordance with protocols approved by the Animal Care and Use Committees of the Ohio State University. TWIK-1^−/−^ mice were created with C57BL/6J genetic background, where the exon 2 gene was deleted (Nie et al., 2005). To confirm this, RT-PCR genotyping was performed by using total mRNA isolated from mice hippocampus (Figures 1A,B). Primers were designed to surround the exon 2 of TWIK-1 mRNA (Accession NM_008430.2): forward, 5′GTGGTCTTCTCGTCCGTG3′; reverse, 5′CCAGGTCTTCGTCCTTGT3′. The designed primers should amplify a 757 and a 362-bp fragment from WT and TWIK-1^−/−^ mice, respectively. Sequencing analysis of the RT-PCR products (GENEWIZ, Inc., USA) further confirmed the deletion of exon 2 (Figure 1C).

All the experiments were performed from the littermates that contained WT, TWIK-1 heterozygote (TWIK-1^+/−^) and TWIK-1^−/−^ male and female mice at postnatal day (P) of 21–28 unless indicated otherwise.

### Quantitative real-time PCR (qRT-PCR) analysis

#### Total RNA extraction from hippocampal tissue

Mice were anesthetized by intraperitoneal injection of 8% chloral hydrate in 0.9% NaCl saline and whole hippocampi were dissected out. After tissue homogenization, total RNA extraction was performed using Qiagen's RNeasy Kit (Valencia, CA), and DNA contamination was prevented by using Qiagen's RNase Free DNase Set (Valencia, CA).

#### Fresh isolation of astrocytes and neurons and RNA extraction

Brain was removed from skull and placed in oxygenated (95% O_2_/5% CO_2_) Ca^2+^-free artificial cerebral spinal fluid (Ca^2+^-free aCSF) (in mM): 125 NaCl, 25 NaHCO_3_, 1.25 NaH_2_PO_4_, 3.5 KCl, 1 MgCl_2_, 1 Na-pyruvate, and 10 glucose, (Zhou and Kimelberg, 2001). Coronal slices of 500 μm thickness were sectioned and transferred to 34°C above aCSF supplemented with 1μM SR-101(Nimmerjahn et al., 2004). After incubation with SR101 for 30 min, the brain slices were transferred to Ca^2+^-free aCSF for another 30 min at room temperature (20–22°C). CA1 regions were dissected out from the brain slices and digested for 25 min with 24U/ml papain in aCSF containing (in mM): 125 NaCl, 25 NaHCO_3_, 1.25 NaH_2_PO_4_, 3.5 KCl, 2 CaCl_2_, 1 MgCl_2_, and 10 glucose, supplemented with 0.8 mg/ml *L*-cysteine. After digestion, the CA1 slices were returned to the Ca^2+^-free-aCSF for recovery for at least 1h. The loosened CA1 slices were gently triturated into cell suspension and then transferred into a recording chamber on a motorized inverted fluorescent microscope (Leica DMIRE2, Leica Microsystems Inc. US). Astrocytes were identified by their positive SR101 fluorescent staining and only cells with typical astrocyte morphology were harvested. Typical pyramidal shaped neurons showing no SR101 staining were collected separately. In each run, thirty cells, both astrocytes and neurons, were collected for each genotype group by a glass electrode (diameter ~10 μm) attached to a micromanipulator. RNA extraction was done by using RNeasy mini kit (Qiagen, California) right after cell harvesting.

#### qRT-PCR

Immediately after RNA extraction, the RNA was converted into cDNA using Applied Biosystem's High Capacity cDNA Reverse Transcription Kit (Grand Islands, NY). The PCR primer pairs for identification of TWIK-1, K_ir_4.1, TREK-1, TWIK-2, TWIK-3, and GAPDH were shown in Table 1. Each primer pair was tested by conventional PCR before qRT-PCR to ensure that the amplicon yielded an anticipated and distinct single band. SYBR® Select Master Mix (Invitrogen, New York) was used and the qRT-PCR was run on a Step One Plus equipment (Life technologies, New York). Each assay was performed in triplicate samples from the same mouse. A minimum of 3 repeats was done for each genotype group, including the neuron control group. For each repetition, a negative control with no template was always present. GAPDH was used as the internal reference and routinely run in parallel with targeted genes. Data were obtained as Ct values (threshold cycle). The expression levels of target genes were expressed as 2^−Δ CT^, where ΔCT was referred to the Ct difference between gene of interest and GAPDH.

**Table 1:** **Primers for qRT-PCR analysis**.

**Target**	**Primer sequence**		**Accession no**.
**gene**			
TWIK-1	F:	AGCAACGCCTCGGGAAAT	NM_008430.2
	R:	GAGGAGGGTGAACGGGAT	
K_ir_4.1	F:	CGCACTTCCTACCTACCG	NM_001039484.1
	R:	GAGATGCCACTTTCACAA	
TREK-1	F:	ACGAAGGAAGAGGTGGGA	NM_001159850.1
	R:	GCACGCTGGAACTTGTCG	
TWIK-2	F:	CTGGTCCTATGGTGATGC	NM_001033525.3
	R:	GTCCCAAAGGTAGAGTGA	
TWIK-3	F:	TTGGGGCTGTGGTGCTTC	NM_010609.2
	R:	GGCAGATCCCAGTTGCTTGT	
GAPDH	F:	AGGTTGTCTCCTGCGACTTCA	NM_008084.2
	R:	GTGGTCCAGGGTTTCTTACTCC	

TWIK, tandem of pore domains in a weak inward rectifying K^+^ channel; TREK, TWIK-related K^+^ channel; K_ir_, inward rectifying K^+^ channel; GAPDH, glyceraldehyde-3-phosphate dehydrogenase. F, forward; R, reverse.

### Preparation of acute hippocampal slices

For slice recording, hippocampal slices were prepared as described previously (Zhou et al., 2009). In brief, after anesthesia, mouse brain was rapidly removed from skull and submerged into ice-cold oxygenated slice cutting solution containing (in mM): 125 NaCl, 3.5 KCl, 25 NaHCO_3_, 1.25 NaH_2_PO_4_, 0.1 CaCl_2_, 3 MgCl_2_, and 10 glucose. Coronal hippocampal slices (250 μm) were cut at 4°C with a Vibratome (Pelco 1500) and transferred to the normal aCSF (osmolality, 295 ± 5 mOsm; pH 7.3–7.4) at room temperature. Slices were kept in aCSF with continuous oxygenation for at least 1h before recording.

### Electrophysiology

To record astrocytes *in situ*, individual hippocampal slice was transferred to the recording chamber with constant perfusion of oxygenated aCSF (2.0 ml/min). The recording chamber is mounted on an Olympus BX51WI microscope and an infrared differential interference contrast (IR-DIC) video camera was used to identify astrocytes located in the CA1 region with the aid of a 40 × water-immersion objective. Whole cell patch-clamp recordings were performed using a MultiClamp 700A amplifier and pClamp 9.2 software (Molecular Devices, Sunnyvale, CA). Borosilicate glass pipettes (outer diameter: 1.5 mm, Warner Instrument) were pulled from a Flaming/Brown Micropipette Puller (Model P-87, Sutter Instrument). The pipettes had a resistance of 5–7 MΩ when filled with gluconate-based pipette solution. A minimum of 2 GΩ seal resistance was required before rupturing the membrane for whole-cell configuration. All the experiments were conducted at room temperature (20 ± 2°C).

All the measurements were made at least 10 min after entering whole-cell recording configuration to allow for adequate solution equilibration. The membrane potential (Vm) was read in “I = 0” mode and membrane resistance (Rm) was measured using the “Membrane test” protocol in the PClamp 9.2 program. Since mature hippocampal astrocytes show very low Rm of about 3 MΩ and an routinely achievable access resistance (Ra) is about 15 MΩ for astrocytes in animals older than P21, a large (~80%) voltage-clamping error occurs in voltage clamp recording (Zhou et al., 2009). Thus in voltage clamp recording, only those recordings with a Ra below 15 MΩ were included for data analysis. In pharmacological whole-cell current analysis, only those recordings where the Ra varied less than 10% were included for data analysis. The liquid junction potential was compensated prior to form the cell-attached mode for all the recordings.

The standard electrode solution contained the following (in mM): 140 K-gluconate, 13.4 Na-gluconate, 0.5 Ca_2_Cl, 1.0 MgCl_2_, 5 EGTA, 10 HEPES, 3 Mg-ATP, and 0.3 Na-GTP (280 ± 5 mOsm). We used 14 mM Na^+^ and 3 mM Cl^−^ as they are physiological in astrocyte (Kelly et al., 2009; Ma et al., 2012a). The Cs^+^-based electrode solution was made by equimolar substitution of K-gluconate with Cs-gluconate. The pH was adjusted to 7.25–7.27 with KOH or CsOH. To determine equilibrium potential of Cs^+^ of astrocyte passive conductance, 3.5 mM extracellular K^+^ was substituted by equimolar Cs^+^ in aCSF solutions. For relative Cs^+^ to K^+^ permeability analysis, modified aCSF solutions containing 70 mM KCl or 70 mM CsCl were made by equimolar substitution of respective ions by NaCl. All the chemicals were purchased from Sigma-Aldrich (St. Louis, MO).

### RT-PCR analysis

Total RNA were extracted from hippocampus, kidney, lung, heart, liver and skeletal muscle by using the same protocol as that for the qRT-PCR experiments noted above. The cDNA synthesis and amplification was performed by Phusion two-step RT-PCR Kit (Thermo Fisher Scientific, Rockford, IL). The primers for detection of TWIK-1 mRNA (Accession NM_008430.2) were designed as: forward 5′GGAAATTGGAATTGGGACT3′, reverse 5′TGCCGATGACAGAGTAGATG 3′, with a predicted product size of 191 bp. Primer for GAPDH mRNA detection (Accession NM_008084.2) were designed as: forward 5′ATTCAACGGCACAGTCAA3′, reverse 5′CTTCTGGGTGGCAGTGAT3′, with a predicted product size of 394 bp.

### Western blot analysis

#### Extraction of total proteins from tissues

Mice were anesthetized as noted and various tissues were rapidly removed and homogenized by a Pro Homogenizer (Oxford, CT) in ice-cold non-denaturing buffer containing 1% Triton X-100, 25 mM Tris-HCl (pH = 7.2), 150 mM NaCl, 1mM EDTA and protease inhibitor cocktails (Sigma-Aldrich, St. Louis, MO). Homogenates were laid on ice for 30 min and then centrifuged (3000×g, 15 min at 4°C). The supernatants were transferred to new tubes and stored as aliquots at −80°C until use.

#### Fractionation of proteins from subcellular regions

Hippocampus and kidney tissues were quickly removed from anesthetized mice and separated into hydrophilic (cytoplasmic) and hydrophobic (membrane) proteins by Mem-PER Eukaryotic Membrane Protein Extraction Kit (Thermo Fisher Scientific, Rockford, IL). In the second fractionation protocol, cytoplasmic, mildly hydrophobic membrane proteins, highly hydrophobic transmembrane proteins, and membrane proteins enriching in lipid raft were separated by using FOCUS™ Global Fractionation Kit (GBioscience, St. Louis, MO). To remove those chemicals that may interfere with the following BCA protein assay and SDS–polyacrylamide gel electrophoresis (SDS-PAGE) analysis, all these fractioned protein samples were cleaned up by SDS-PAGE Sample Prep Kit (Thermo Fisher Scientific, Rockford, IL).

#### SDS-page and immunoblotting

Protein concentration was determined with the BCA protein assay kit (Thermo Fisher Scientific, Rockford, IL). Samples were mixed with a 5× reducing loading buffer containing 100 mM DTT (Thermo Fisher Scientific, Rockford, IL) and heated at 95°C for 5 min. Equal amounts of proteins (25 μ g/lane) were separated on a 4–12% tris-glycine gel (Bio-Rad, Hercules, CA, USA) and subsequently transferred to a nitrocellulose membrane (Micron Separations Inc., Westborough, MA). The membranes were blocked with 5% non-fat milk/TBST (Tris-buffered saline with 0.05% Tween 20) for 1h at room temperature. The membranes were incubated with anti-TWIK-1 antibodies (1:2000, Alomone Labs, Jerusalem, Israel) at 4°C overnight. After secondary antibody incubation (Jackson ImmunoResearch Laboratories, Maine, US), immunoreactivity was detected with an enhanced chemiluminescent detection (Thermo Fisher Scientific, Rockford, IL). Blots were scanned and quantified by Quantity One software (Bio-Rad, Hercules, CA, USA). After detecting TWIK-1 immunoreactivity, the original membranes were stripped with stripping buffer (0.4 M Glycine, 0.2% SDS and 2% Tween 20, pH 2.0) and re-probed with the following primary antibodies sequentially to determine the quality of protein fractionation: anti-caveolin-1 (CAV-1) (1:250; Abgent, San Diego, CA), anti-Na^+^/K^+^ ATPase alpha 2(+) polypeptide (ATP1α2) (1:1000; Abgent, San Diego, CA), anti-K_ir_4.1 (1:600, Alomone Labs, Jerusalem, Israel), anti-glyceraldehyde-3-phosphate dehydrogenase (GAPDH) (1:8000, Sigma-Aldrich, St. Louis, MO), or anti-glial fibrillary acidic protein (GFAP) (1:500, DAKO, Carpinteria, CA).

### Data analysis

Rectification index (RI) was used to determine the selective impact of TWIK-1 gene deletion on astrocyte passive conductance. RI was calculated by dividing the current amplitudes induced by +20 mV (y1) over −180 mV (y2) (Figure 3).

(1) $$RI=y1/y2=I_{20\text{mV}}/I_{-180\text{mV}}$$

The relative Cs^+^ to K^+^ permeability (*P*_Cs_/*P*_K_) was determined by the relative shift in reversal potentials when K^+^ in the bath solution was replaced by equimolar Cs^+^ and calculated according to the following equation:

(2) $$\frac{P_{\text{Cs}}}{P_{\text{K}}}=\text{exp ​}\left[\left({\text{E}}_{\text{Cs}}-{\text{E}}_{\text{K}}\right)\text{​}/\text{​}(\text{RT/zF})\right]$$

E_K_ and E_Cs_ were the equilibrium reversal potential of K^+^ and Cs^+^, respectively.

The patch clamp recording data were analyzed by Clampfit 9.0 (Molecular Devices, Sunnyvale, CA) and Origin 8.0 (OriginLab Corporation, MA). Data are presented as means ± SEM. Student's unpaired *t*-test was used for statistical analysis of two independent samples. For multiple comparison tests, WT was used as control group, and TWIK-1^+/−^ and TWIK-1^−/−^ were compared to against it. Thus, One-Way ANOVA followed by Dunnett's test were combined for these tests. Significance level was set at *P* < 0.05.

## Results

### TWIK-1 knockout mice

In genotyping analysis, RT-PCR amplification of mRNAs isolated from TWIK-1^−/−^ and TWIK-1^+/−^ mice hippocampus revealed an anticipated truncated TWIK-1 transcript with a size consistent with a total deletion of exon 2 of the TWIK-1 gene (Figures 1A,B) (Nie et al., 2005). Sequencing analysis further confirmed a deletion of 396 nucleotides of exon 2 encoding amino acid residues 119–250, including the two pore-forming domains of TWIK-1 (Figure 1C) (Miller and Long, 2012). Thus TWIK-1 channel activity is absent in TWIK-1^−/−^ mice.

As reported previously, the growth, fertility and gross anatomy of TWIK-1^−/−^ mice did not differ from their age- and gender-matched wild type mice, and the offspring from heterozygote mating followed a Mendelian ratio (Nie et al., 2005).

### TWIK-1 gene deletion does not alter the mRNA expression of major astrocyte k^+^ channels

To clarify whether TWIK-1 gene knockout interferes with the expression of the other major astrocyte K^+^ channels, or produces compensation for the loss of TWIK-1, we compared the expression levels of mRNAs for a group of K^+^ channels that were selected based on the following considerations. First, we included the three major astrocyte K^+^ channels known to expressed with the relative levels of TWIK-1 > K_ir_4.1> TREK-1 in isolated cortical astrocytes (Cahoy et al., 2008) and freshly isolated hippocampal astrocytes (Seifert et al., 2009). Second, we selected another two K2Ps in the TWIK subfamily, TWIK-2 and TWIK-3; the former conducts weakly inward rectifying K^+^ currents, whereas the latter does not produce measurable functional currents in heterologous expression systems (Enyedi and Czirjak, 2010).

**Figure 2:**
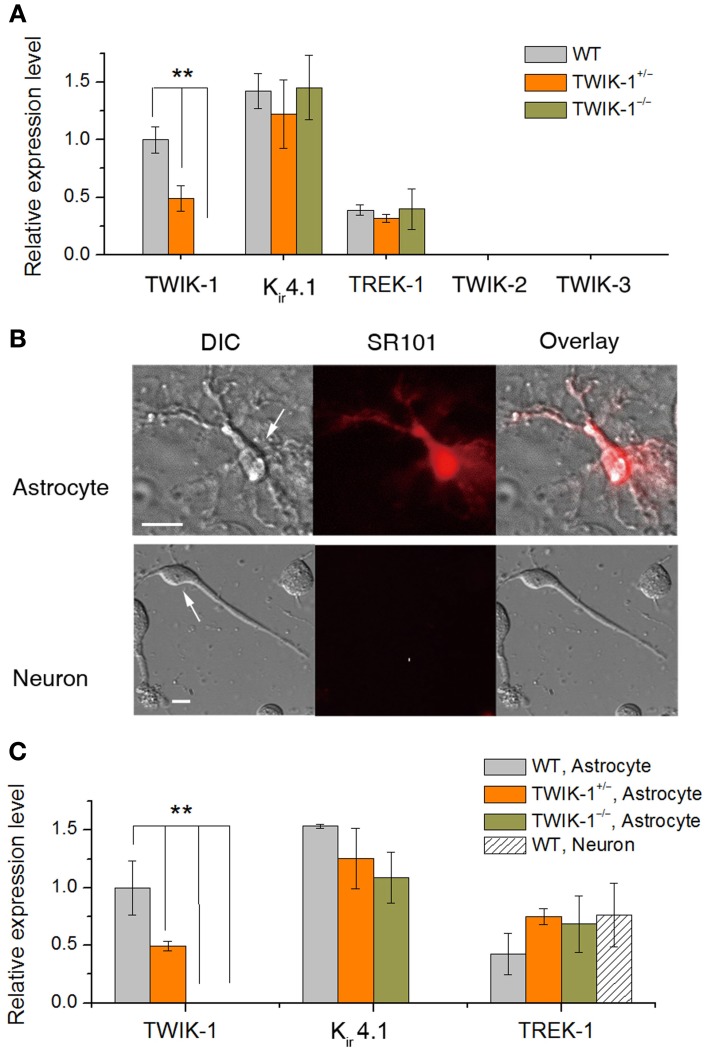
**TWIK-1 deletion does not alter the expression pattern of astrocyte K^+^ channels. (A)** qRT-PCR results of the relative quantity of TWIK-1, K_ir_4.1, TREK-1, TWIK-2, and TWIK-3 from the total mRNA isolated from mice hippocampus. The expression levels of TWIK-1 mRNAs were reduced to half and hundred percent in TWIK-1^+/−^ and TWIK-1^−/−^ mice, respectively. **(B)** Morphology of freshly isolated astrocyte and neuron from mice hippocampus (DIC). Scale bar: 10 μm. These cells were harvested separately for qRT-PCR analysis. Astrocytes were selected based on their positive SR101 staining (middle up panel), and SR101 staining was completely devoid in isolated pyramidal neurons (middle bottom panel). **(C)** The expression pattern of TWIK-1, K_ir_4.1 and TREK-1 of isolated astrocytes resembled that of the hippocampal tissues **(A)**. The expression of TWIK-1 and K_ir_4.1 appeared to be astrocytic, while TREK-1 was both astrocytic and neuronal. Data were normalized to the expression level of TWIK-1 mRNA in WT. In each genotype group, 30 isolated astrocytes or neurons were harvested from 3 different mice and analyzed separately to obtain a mean ± SEM (*n* = 3). ^**^P < 0.01.

In WT hippocampal tissue, qRT-PCR revealed TWIK-1, K_ir_4.1 and TREK-1 mRNA expression in mice with relative abundance K_ir_4.1> TWIK-1> TREK-1 (Figure 2A), but neither TWIK-2 nor TWIK-3 was detected. The amount of TWIK-1 mRNA was reduced by around 50% in TWIK-1^+/−^ and 100% in TWIK-1^−/−^ mice. In contrast, in TWIK-1^+/−^ and TWIK-1^−/−^ mice, the expression levels and the relative abundance of K_ir_4.1 and TREK-1 were not altered compared to the wild type (Figure 2A). Thus TWIK-1 gene deletion did not result in any apparent compensatory change in these candidate K^+^ channels.

To verify that TWIK-1 expression is astrocytic at the cellular level, freshly isolated hippocampal astrocytes and neurons were harvested as described previously (Zhou and Kimelberg, 2001) and qRT-PCR analysis was repeated using freshly isolated astrocytes and neurons separately. Astrocytes were selected based on their characteristic morphology and positive staining for SR101. Hippocampal neurons were harvested based on their distinctive morphology and absence of SR101 staining (Figure 2B). While TREK-1 could be detected in both astrocytes and neurons, K_ir_4.1 and TWIK-1 were detected only in astrocytes, indicating specific localization to astrocytes in this brain region. In wild type astrocytes, the relative levels of mRNA for these channels followed the same order as for hippocampal tissue described above. TWIK-1 mRNAs were abundant in astrocytes in WT mice, reduced to around 50% in TWIK-1^+/−^ and completely absent in TWIK-1^−/−^ mice. Similarly, the expression of K_ir_4.1 and TREK-1 was not altered among three genotype groups (Figure 2C).

In summary, TWIK-1 exhibits a cell type specific expression in hippocampal astrocytes, and at transcript level TWIK-1 gene knockout did not alter the expression pattern of other astrocytic K^+^ channels that have the potential to compensate for changes in astrocyte passive conductance.

### Membrane properties of hippocampal passive astrocytes in TWIK-1 knockout mice

**Figure 3:**
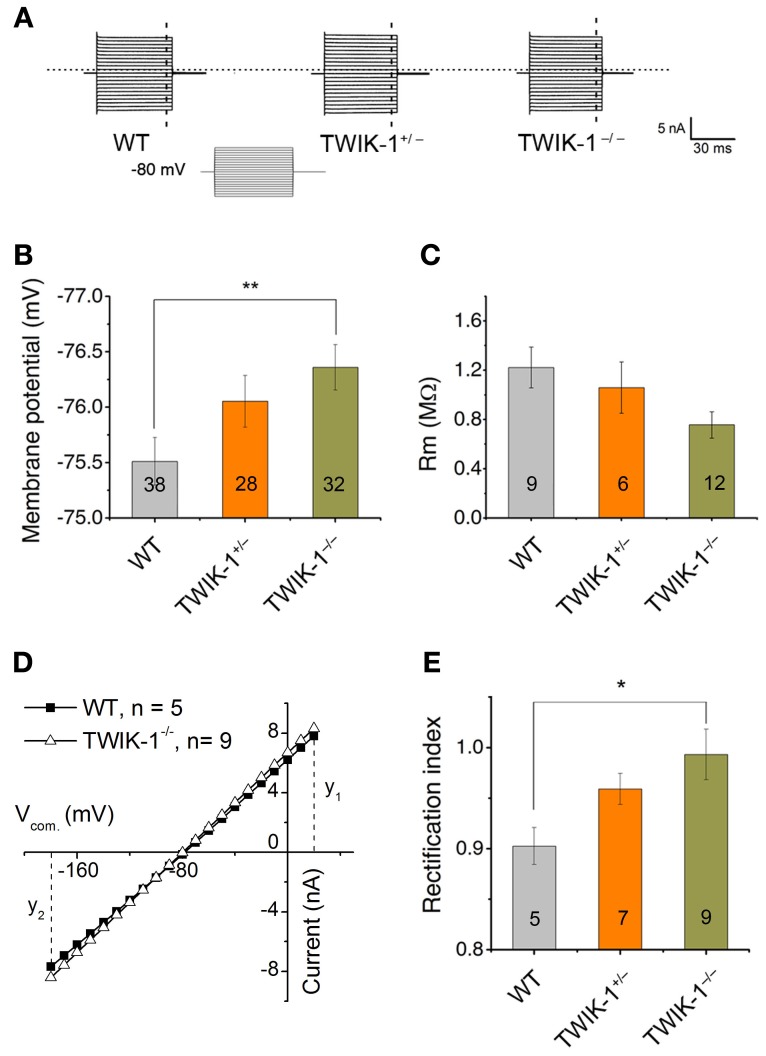
**TWIK-1 gene deletion alters the membrane properties of astrocytes. (A)** Representative whole-cell currents of three genotypes. Cells were held at −80 mV at resting and the membrane currents were induced by command voltages from −180 to +20 mV with 10 mV increment (*inset*, command voltages, Vcom). Astrocytes in all genotypes showed a similar linear I–V relationship membrane conductance. **(B), (C)** TWIK-1 gene deletion hyperpolarized astrocyte membrane potential (Vm, **B**), but did not significantly alter the membrane resistance (Rm, **C**). **(D)** To construct I–V curves, the current amplitudes from the dashed vertical lines in **(A)** were plotted against their corresponding Vcom. The current amplitude at y1 (V_20 mV_) and y2 (V_-180 mV_) were used to calculate RI according to equation [1] (see Methods). **(E)** The RI values shifted from weakly inward rectification in WT to close to linear in TWIK-1^−/−^ astrocytes in a gene dependent manner. Data are shown as mean ± SEM, ^*^p < 0.05, ^**^p < 0.01.

At the mRNA expression level, TWIK-1 appears to be the most abundant K^+^ channels in isolated cortical astrocytes (Cahoy et al., 2008). In the present study, TWIK-1 mRNA also appears to express highly in isolated hippocampal astrocytes. To test the functional contribution of TWIK-1 to passive conductance, we compared whole-cell currents, resting membrane potential (Vm) and membrane resistance (Rm) of mature CA1 hippocampal astrocytes in slices among TWIK-1^+/+^, TWIK-1^+/−^and TWIK-1^−/−^ mice (Figure 3). As TWIK-1 mRNAs (Cahoy et al., 2008) and proteins (Figure 5D) expression increase during postnatal development, mature astrocytes from animals older than P21 were used in this study. Unexpectedly, TWIK-1 gene knockout did not lead to a substantial loss of passive conductance in astrocytes (Figure 3A). However, the Vm value of astrocytes shifted to hyperpolarized potentials in a TWIK-1 gene dependent manner; −75.51 ± 0.21 mV (*n* = 38) in WT, −76.05 ± 0.23 mV (*n* = 28) in TWIK-1^+/−^, and −76.35 ± 0.20 mV (*n* = 32) in TWIK-1^−/−^ (Figure 3B). Despite the small value of this Vm shift, ΔVm = 0.84 mV, the difference between WT and TWIK-1^−/−^ was statistically significant (*P* = 0.011, Figure 3B). Elimination of TWIK-1 would anticipate to increasing Rm. However, the Rm apparently decreased in a TWIK-1 gene dependent manner, WT (1.22 ± 0.17 MΩ, *n* = 9), TWIK-1^+/−^ (1.06 ± 0.21 MΩ, *n* = 6), and TWIK-1^−/−^ (0.76 ± 0.11 MΩ, *n* = 12), but the difference was not statistically significant (Figure 3C, *P* = 0.052) and the mechanism accounting for this change is yet unknown.

The TWIK-1 K^+^ channel is weakly inwardly rectifying as a result of rapid current inactivation at depolarized potentials (Lesage et al., 1996). To determine whether TWIK-1 contributes to the rectification characteristic of passive conductance, we used the rectification index (RI, see Methods) to explore a potential impact of TWIK-1 deletion on passive conductance. Astrocytes in WT mice showed weak inward rectification with a RI of 0.90 ± 0.018 (*n* = 5), and this rectification was significantly straightened to a close to linear I–V relation in TWIK-1^−/−^ mice (RI = 0.99 ± 0.03, *n* = 9, *P* = 0.035, Figures 3D–E).

**Figure 4:**
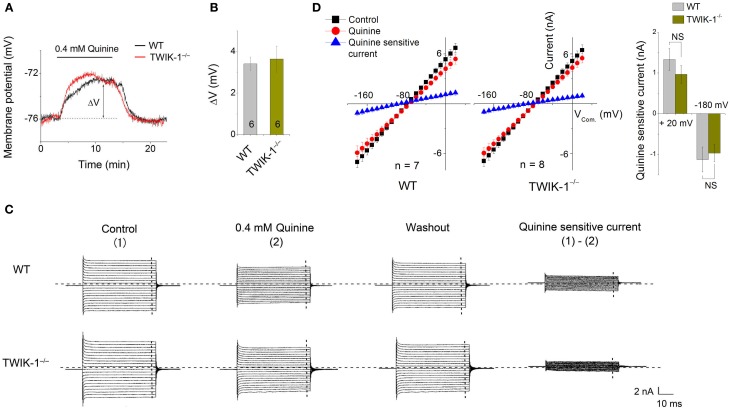
**Effects of quinine on astrocyte membrane potential and passive conductance in WT and TWIK-1^−/−^ mice. (A)** 0.4 mM quinine induced comparable amplitude of Vm depolarization in both WT and TWIK-1^−/−^ astrocyte. ΔV indicates the plateau amplitude of Vm depolarization at 8 min of quinine application. **(B)** Summary of ΔV from both genotypes. **(C)** The representative whole-cell passive currents before and after 8 min 0.4 mM quinine application. The Vcom protocol shown in Figure 3A was also used here for membrane current induction. I–V relationships were shown in **(D)**. The quinine sensitive currents were obtained from sweep subtraction, which were linear I–V relationship in both genotypes. Data are shown as mean ± SEM.

Quinine, a non-specific K^+^ channel inhibitor, has been shown as an effective TWIK-1 channel inhibitor with an IC_50_ of ~85 μ M (Zhou et al., 2009). When 0.4 mM quinine was bath applied, the Vm depolarization in astrocytes was comparable between WT (3.41 ± 0.32 mV, *n* = 3) and TWIK-1^−/−^ (3.64 ± 0.62 mV, *n* = 3) mice (*P* > 0.05, Figures 4A,B). Similarly, there was no significant difference in the amplitude of subtracted quinine sensitive currents between WT (*n* = 7) and TWIK-1^−/−^ mice (*n* = 8) (Figures 4C–D). Specifically, the subtracted quinine currents were −1.12 ± 0.31 nA and −0.97 ± 0.21 nA at −180 mV command voltage (*P* = 0.674), and 1.33 ± 0.27 nA and 0.97 ± 0.22 nA at +20 mV command voltage (*P* = 0.312) for WT and TWIK-1^−/−^, respectively. These results suggest that TWIK-1 channels, presumably as part of the quinine sensitive channels, make a minor contribution to membrane potential and passive current profile in mature astrocytes.

**Figure 5:**
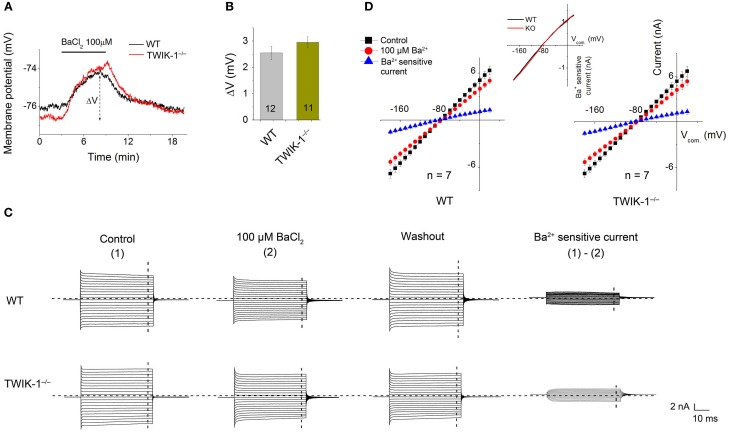
**Ba^2+^ at micromolar concentration does not eliminate the inward component of passive conductance in TWIK-1^−/−^ mice. (A)** 100 μ M BaCl_2_ induced similar Vm depolarization in WT and TWIK-1^−/−^ astrocytes. Δ V indicates the plateau Vm depolarization at 5 min in BaCl_2_. **(B)** 100 μ M BaCl_2_ induced Δ V was not differed between WT and TWIK-1^−/−^ astrocytes. **(C)** The representative whole cell passive currents before and after 5 min 100 μ M BaCl_2_ application. I–V relationships were shown in **(D)**. The Ba^2+^ sensitive currents were obtained from sweep subtraction. The Ba^2+^ sensitive currents were shown in expanded y-axis in the inset in **(D)** that showed a moderate inward rectification in both WT, RI = 0.86, and TWIK-1^−/−^, RI = 0.87, astrocytes. Data are shown as mean ± SEM. Numbers of experiments are given in the bars.

To ensure that, at functional level, K_ir_4.1 does not compensate for the inward whole-cell current in TWIK-1^−/−^ mice, we compared micromole concentration Ba^2+^ effect on Vm and whole-cell passive conductance between astrocytes in WT and TWIK-1^−/−^ mice. 100 μM Ba^2+^ is known to inhibit K_ir_4.1 channel currents fully with an IC_50_ of 3.5 μM (Ransom and Sontheimer, 1995). However, Ba^2+^ effect on Vm depolarization did not differ significantly between WT (2.55 ± 0.25 mV, *n* = 12), and TWIK-1^−/−^ mice (2.95 ± 0.21 mV, *n* = 11; Figures 5A,B). And 100 μM Ba^2+^ also showed a similar inhibitory effect on passive conductance (Figures 5C,D). The results indicate that the functional K_ir_4.1 currents are not altered in TWIK-1^−/−^ mice.

In summary, TWIK-1 gene knockout produces perceptible changes in astrocyte Vm and RI. However, the overall passive conductance and Vm are not altered in a major way, suggesting that other K^+^ channels make a more substantial contribution to the basic electrophysiological properties of mature astrocytes.

### TWIK-1 knockout decreases the Cs^+^ permeability of passive conductance

**Figure 6:**
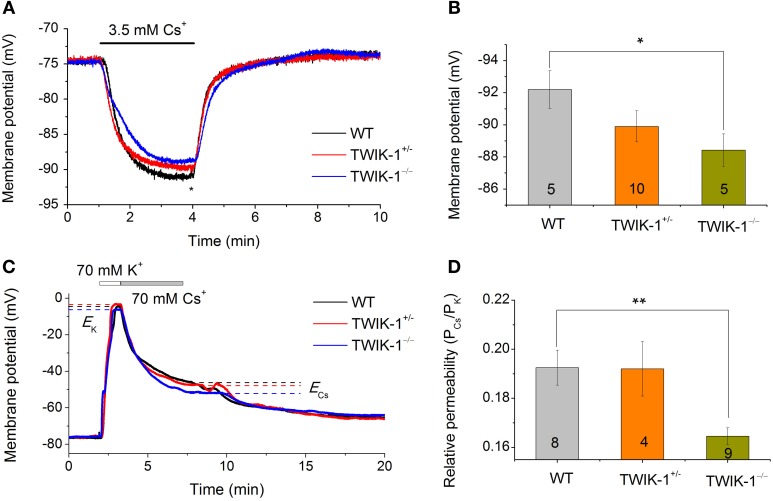
**TWIK-1 gene deletion decreases the relative Cs^+^ to K^+^ permeability of passive conductance. (A)** Astrocyte membrane potential (Vm) approaches to Cs^+^ equilibrium potential (*E*_Cs_) in Cs^+^ -based pipette and bath solutions. Switch extracellular 3.5 mM K^+^ to 3.5 mM Cs^+^ resulted in a rapid negative Vm shift in all three genotypes with a net negative shift of around −10 mV at the plateau in 2–3 min. The plateau Vm values at the time point indicated by “*” were summarized in **(B)**. The Cs^+^ established Vm showed a TWIK-1 gene dependent positive shift and the difference between WT and TWIK-1^−/−^ mice was statistically significant. **(C)** Representative whole-cell membrane potential recording traces, with bath solutions switched from normal aCSF containing 3.5 mM K^+^ to solutions containing, in sequence, 70 mM K^+^ and 70 mM Cs^+^. *E*_Cs_ and *E*_K_ are the equilibrium potential of Cs^+^ and K^+^ at 70 mM, respectively. **(D)** The relative Cs^+^ to K^+^ permeability (*P*_Cs_/*P*_K_) values for three genotypes; the *P*_Cs_/*P*_K_ of TWIK-1^−/−^ mice was significantly lower than that of the TWIK-1. Data are shown as mean ± SEM. Numbers of experiments are given within the bars. ^*^p < 0.05, ^**^p < 0.01.

While Cs^+^ blocks various K^+^ channels, including the classical voltage-gated K^+^ channels and inward rectifier K^+^ channels (Hille, 2001), TWIK-1 is one of the K2Ps known to be highly permeable to Cs^+^ (Zhou et al., 2009; Ma et al., 2011). The passive conductance of rat hippocampal astrocytes exhibits a distinctively high Cs^+^ to K^+^ relative permeability ratio (*P*_Cs_/*P*_K_) of 0.42 (Zhou et al., 2009). To determine the relative contribution of TWIK-1 to the high Cs^+^ permeability in astrocytes, two experiments were carried out. First, we used Cs^+^-based recording solutions to determine the reversal potential of the Cs^+^ mediated conductance. Cells were recorded with a Cs^+^- based electrode solution first in normal aCSF with 3.5 mM K^+^, then in 3.5 mM Cs^+^ in replace of K^+^. In all three animal groups, this bath solution switch produced a large Vm hyperpolarization that reached a plateau Vm in ~2.5 min (Figure 6A). Overall, the Cs^+^ established Vm was very close to the Cs^+^ equilibrium potential (*E*_Cs_= −93 mV), but astrocytes from TWIK-1^−/−^ mice exhibited a significantly less negative plateau Vm compared to those from WT mice (−88.02 ± 0.79 mV in TWIK-1^−/−^, *n* = 5 *vs.* −92.2 ± 1.18 mV in TWIK-1^+/+^, *n* = 5; *P* = 0.046; Figure 6B), indicating that TWIK-1 channels contribute to a significant proportion of the Cs^+^ permeable channels. In the total absence of K^+^ in the recording solutions, Cs^+^ still mediated a passive conductance in all three genotype groups. Nevertheless, the Cs^+^-mediated passive membrane conductance exhibited a weak outward rectification and this was not affected by TWIK-1 gene knockout (WT 1.09 ± 0.04, *n* = 5, *vs*. TWIK-1^−/−^ 1.09 ± 0.03, *n* = 5, *P* = 0.661). Taken together, TWIK-1 contributes to a portion of the functional Cs^+^-permeable K^+^ channels, but other Cs^+^-permeable K^+^ channels are likely to contribute to a greater degree to the astrocyte passive conductance.

In the second set of experiments, we examined the *P*_Cs_/*P*_K_ ratio among three genotype groups using a procedure described previously (Zhou et al., 2009). In brief, the Vm of astrocytes was first recorded in normal aCSF with 3.5 mM K^+^. The bath perfusate was then switched to 70 mM K^+^ and 70 mM Cs^+^ solutions sequentially (Figure 6C). The equilibrium Vm in 70 mM K^+^ and 70 mM Cs^+^were then used to calculate the *P*_Cs_/*P*_K_ ratio (Equ. 2, Methods). The *P*_Cs_/*P*_K_ ratio decreased significantly by 14.5% in TWIK-1^−/−^ mice compared to WT (0.193 ± 0.007 in TWIK-1^+/+^, *n* = 8; 0.192 ± 0.11, *n* = 4 in TWIK-1^+/−^; 0.165 ± 0.003 in TWIK-1^−/−^, *n* = 9). The difference between the WT and TWIK-1^−/−^ groups was statistically significant (*P* = 0.006, Figure 6D).

In summary, TWIK-1 is one component of the Cs^+^-permeable channels that functionally contribute to the passive conductance of mature astrocytes. Of note, the overall *P*_Cs_/*P*_K_ ratio in mice appears to be markedly lower than that of the rats described before (Zhou et al., 2009).

### TWIK-1 channels are preferentially retained in cytoplasm in astrocytes

Because TWIK-1 gene knockout only mildly affects the membrane potential and passive conductance, we explored further the mechanism underlying this observation. In kidney tubular epithelial cells, TWIK-1 presented mainly as a cytoplasmic protein (Decressac et al., 2004; Nie et al., 2005). We therefore tested whether this is also the case in astrocytes using subcellular protein fractionation and western blotting.

**Figure 7:**
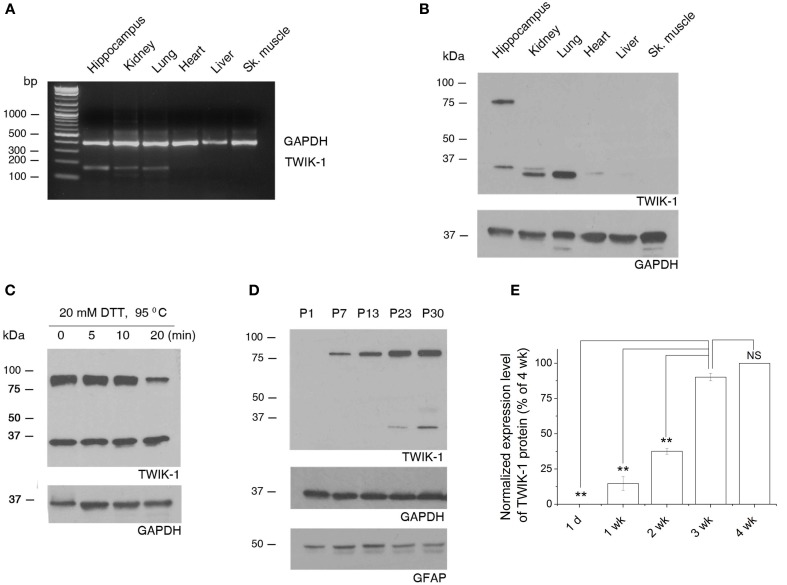
**Tissue specific expression of TWIK-1 mRNA and proteins and age-dependent up-regulation of TWIK-1 in hippocampus. (A)** RT-PCR analysis of TWIK-1 mRNA expression in various adult mouse tissues. TWIK-1 mRNA expression is high in hippocampus, kidney and lung, but barely detectable from heart, liver and skeletal (Sk.) muscle. GAPDH was measured as internal control. The PCR product size for TWIK-1 and GAPDH gene were 191 bp and 394 bp, respectively. **(B)** Representative Western blot results analyzed from various tissues noted above. Note that TWIK-1 protein expression followed exactly the same tissue-specific pattern of TWIK-1 mRNA. **(C)** When treating the hippocampal tissue samples with the reducing agent dithiothreitol (DTT, 20 mM) at different times, the ratio of TWIK-1 dimer versus monomer decreased in a time-dependent manner. **(D)** A representative blotting result of TWIK-1 proteins from mice hippocampus of different ages as indicated. Both GAPDH (36 kDa) and GFAP (50 kDa) were the loading controls from the same blots. **(E)** Quantitation of TWIK-1 expression levels from different ages as shown in **(D)**. Data were normalized to the levels of 3 week (wk.), *n* = 3. ^*^p < 0.05, ^**^p < 0.01.

We first explored the tissue specific expression of TWIK-1 with the total mRNAs and proteins extracted from hippocampus, kidney, lung, heart, liver, and skeletal muscle of wild type mice. Both TWIK-1 mRNA and protein are expressed highly in hippocampus, kidney and lung, weakly in heart and are barely detectable in liver and skeletal muscle (Figures 7A,B), which corresponded well with the tissue specific expression pattern of TWIK-1 reported previously (Lesage et al., 1997).

Functional TWIK-1 homodimers are formed by an inter-monomer disulfide bond at cysteine 69 (C69) (Lesage et al., 1997; Miller and Long, 2012). Interestingly, under the same protein denaturing and reducing conditions, both TWIK-1 monomer (~35 kDa) and dimer (~80 kDa) were seen in hippocampus, but the monomer was the only form detected in lung and kidney (Figure 7B, *n* = 4). This suggests that different mechanisms may be involved in stabilizing the conformation of TWIK-1 channels in different tissues. The detection of both TWIK-1 monomer and dimer with similar size in rat hippocampus has been reported recently (Pivonkova et al., 2010). Extending DTT incubation time resulted in a time-dependent decrease in the ratio of dimer versus monomer (Figure 7C), confirming that both bands were TWIK-1 channel proteins. As the band density is proportional to the amount of TWIK-1 monomers, we included the signal from both 35 and 80 kDa bands for total TWIK-1 protein quantification.

Because TWIK-1 mRNA increases with age (Cahoy et al., 2008), we examined protein levels by western blot analysis of hippocampal proteins extracted at different ages. Consistently, TWIK-1 protein was absent at postnatal day 1 (P1), increased with age and reached a final expression level after the third postnatal week (Figures 7D,E).

**Figure 8:**
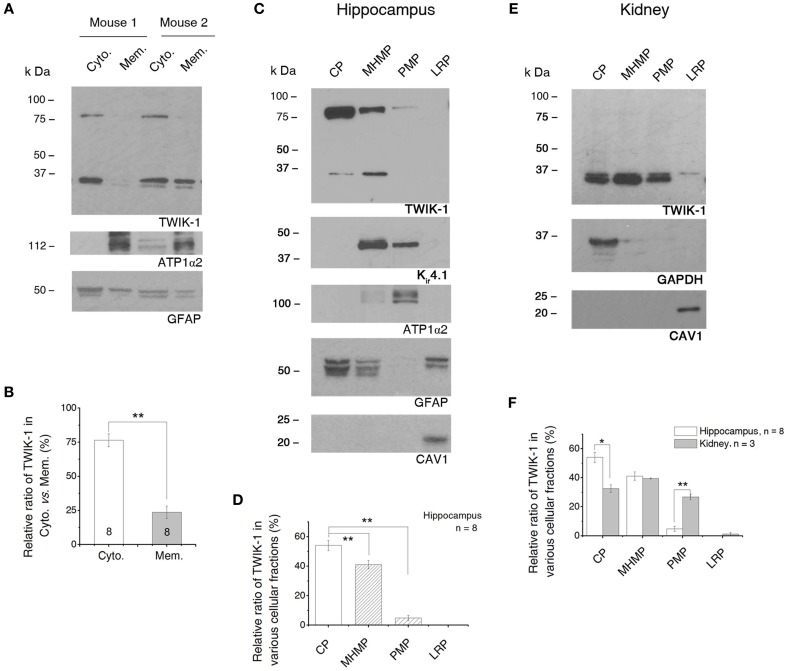
**TWIK-1 channels are predominantly located in cytoplasm. (A)** Western blot results of TWIK-1 location in cytoplasmic *vs*. membrane proteins of two mice hippocampus. GFAP (50 kDa) and ATP1α2 (112 kDa) were markers for cytoplasmic and membrane fractions, respectively. Note that TWIK-1 dimer and monomer were mainly located in cytoplasmic fraction in both mice. **(B)** A bar graph showing the relative ratio of TWIK-1 proteins located in cytoplasmic and membrane fractions. **(C)** A representative blotting result from the second fractionation method (see Methods). Total protein from hippocampus was fractionated into cytoplasmic proteins (CP), mildly hydrophobic membrane proteins (MHMP), plasma membrane protein (PMP) and lipid raft proteins (LRP). GFAP, ATP1α2 and CAV1 (21 kDa) were markers for CP, PMP and LRP, respectively. TWIK-1 also expressed highly in cytoplasm, relative less in MHMP and very low in PMP fraction. K_ir_4.1 (42 kDa), another glial specific K^+^ channel, was detected from both of the MHMP and PMP as anticipated. **(D)** Quantitation of TWIK-1 subcellular location in the four subcellular regions noted above (*n* = 8). **(E)** A representative blot with the second fractionation method in **(C)** from kidney proteins. GAPDH and CAV1 were used as cytoplasm and lipid raft markers, respectively. The amount of TWIK-1 channels in PMP fraction was significantly higher in kidney than that of the hippocampus. **(F)** Comparison of the subcellular location of TWIK-1 between hippocampus (*n* = 8) and kidney (*n* = 3). The blots shown in **(A)**, **(C)** and **(E)** were all first incubated with anti-TWIK-1 antibody and then re-probed with the rest of primary antibodies sequentially after the original membranes were stripped with stripping buffer. ^*^p < 0.05, ^**^p < 0.01.

To gain insight into the subcellular distribution pattern of TWIK-1, cytoplasmic and membrane proteins were extracted from hippocampus for fractionation and western blot analysis (Thermo Scientific protocol, see Methods; Figure 8A). Antibodies against GFAP and ATP1α2 were used as astrocyte-specific cytoplasmic and membrane markers (Eng et al., 2000; Dinuzzo et al., 2012). In eight tests from different mice hippocampal samples, TWIK-1 monomer and dimer always co-appeared in the cytoplasmic fraction, while only monomer was detected in membrane fraction in 4/8 experiments (Figure 8A). Quantitatively, only 23.6 ± 4.65% of TWIK-1 protein was distributed in the membrane fraction in contrast to the majority (76.4 ± 4.65%) in the cytoplasmic fraction (*n* = 8, *P* = 0.01; Figure 8B).

To delineate the subcellular distribution more precisely, a second fractionation protocol was introduced (GBioscience protocol, see Methods). The initial step of this protocol separated the soluble proteins from insoluble proteins. Then the insoluble proteins were further fractionated into the mildly hydrophobic membrane proteins (such as peripheral membrane proteins), highly hydrophobic transmembrane proteins (plasma membrane-enriched fraction) and membrane proteins enriched in lipid rafts. Because the protocol uses a relatively low centrifugation speed (20,000×g), therefore the “soluble” protein fraction constitutes of a mixture of cytosol and low-density intracellular vesicles (such as endosome, *trans* Golgi net and transport vesicles) (Bonifacino et al., 2004). Thus the term “cytoplasmic fraction” is used to cover the mixed protein content in this fraction. We confirmed the subcellular localization of each of these fractions by blotting them with antibodies against ATP1α2 (plasma membrane-specific protein), GFAP (astrocyte cytoplasmic-specific protein), GAPDH (cytosolic-specific protein), and caveolin-1 (CAV1, lipid raft membrane marker) (Juhaszova and Blaustein, 1997; Dinuzzo et al., 2012) (Figures 8C,E). TWIK-1 appeared predominantly in cytoplasmic fraction (54.07 ± 3.45%, *n* = 8), with lower levels in the mildly hydrophobic membrane protein fraction (41.11 ± 2.90%, *n* = 8; *P* = 0.007 as compared with cytoplasmic fraction), even less in the plasma membrane fraction (4.82 ± 1.77%, *n* = 8; *P* = 0.0001 as compared with cytoplasmic fraction) and was totally absent from the lipid raft fraction (Figure 8D).

K_ir_4.1 channels are known to present on the surface membrane and located on astrocytic processes surrounding synapses and blood vessels (Higashi et al., 2001). Thus we compared K_ir_4.1 expression with that of TWIK-1 in the same membrane blots first used for TWIK-1 detection. As expected, a much stronger K_ir_4.1 signal was detected in plasma membrane fractions (Figure 8C). Notably, K_ir_4.1 was barely detectable in the cytoplasmic fraction. In addition, K_ir_4.1 was also detected in the mildly hydrophobic membrane protein fractions, which may result from the direct binding of K_ir_4.1 channels with alpha-syntrophin, a typical peripheral protein, in mouse astrocytes (Connors et al., 2004).

Localization of TWIK-1 within the cytoplasm has been shown in an immunocytochemical study using kidney cells (Nie et al., 2005), but no quantitative information was provided. To determine if a similar subcellular TWIK-1 distribution pattern occurs in other tissue, we next repeated the second fractionation procedure with the samples from kidney. Overall, TWIK-1 distributed in kidney cells with a similar pattern as in the hippocampus (Figure 8E), but with the following differences: (1) a large amount of TWIK-1 (32.52 ± 3.68%, *n* = 3) was localized to the cytoplasmic fraction, but this was significantly less than in the hippocampus (*P* = 0.006, Figure 8F); (2) while levels of TWIK-1 in the mildly hydrophobic membrane fraction were comparable to those in hippocampus, a significantly higher amount of TWIK-1 appeared in the plasma membrane fraction compared to hippocampus (26.75 ± 2.71%, *P* = 0.0001, Figure 8F); (3) as in the results shown in Figure 7B, no dimer was detected from kidney tissue in any of the fractions.

In summary, TWIK-1 is mainly located in cytoplasm of astrocyte. Additionally, the relative amount of TWIK-1 in plasma membrane fractions is substantially lower in hippocampal astrocytes than in other TWIK-1 highly expressing tissues, such as kidney.

## Discussion

The present study provides the first direct evidence for the actual functional contributions of TWIK-1 channels to the electrophysiological properties of mature astrocytes. Because the majority of TWIK-1 protein appears to be localized in intracellular compartments, TWIK-1 contributes only mildly to the overall astrocyte passive K^+^ conductance.

### Contribution of TWIK-1 to astrocyte passive conductance

In mouse, TWIK-1 mRNA is particularly abundant in brain, kidney and lung, but is only weakly expressed in heart, liver and skeletal muscle (Lesage et al., 1997). Our RT-PCR and western blot experiments revealed a similar expression pattern of TWIK-1 in these tissues (Figures 7A,B). In line with two previous reports (Cahoy et al., 2008; Zhou et al., 2009), we further confirmed the astrocytic expression of TWIK-1 by comparing TWIK-1 mRNA from isolated hippocampal astrocytes versus neurons. Accordingly, it is reasonable to infer that the western blot results reflect TWIK-1 expression and subcellular distribution in hippocampal astrocytes.

We show that TWIK-1 gene knockout induced a hyperpolarized membrane potential (Figure 3B), a shift in the rectification index (Figure 3D) and decrease in the *P*_Cs_/*P*_K_ ratio in TWIK-1^−/−^ mice (Figure 6). All these changes are consistent with the reported biophysical properties of TWIK-1 channels, and all these changes occurred to a lesser degree in heterozygous TWIK-1^+/−^ mice. Thus these results support that TWIK-1 channels contribute functionally to a certain degree to the astrocyte passive conductance. Nevertheless, the overall whole-cell passive conductance was not altered significantly in TWIK-1 KO astrocytes. Consistent with this notion, the non-specific TWIK-1 inhibitor quinine also failed to detect an apparent difference in terms of inhibition of passive conductance and Vm depolarization between WT and KO astrocyte.

At the time TWIK-1 was first cloned, large amount of TWIK-1 cDNA injection induced only small currents in oocytes (Lesage et al., 1996), and even less whole-cell current in transfected CHO cells (Rajan et al., 2005). A point mutation in carboxyl-terminus, K274E, created a TWIK-1 mutant that generates large K^+^ currents (Rajan et al., 2005; Ma et al., 2011, 2012b). More recently, substitution of three glycines in the M2 segment (L146G, A151G, and V153G) led to a 16-fold increase in TWIK-1 currents and conversion of channel conductance from weak inward rectification to GHK outward rectification (Chatelain et al., 2012). Thus, in its “wild-type” resting state, TWIK-1 appears to be a quiescent channel and this may in part explain a mild contribution of TWIK-1 to the total astrocyte passive conductance.

### Subcellular location of TWIK-1 protein in hippocampal astrocytes

Previous immunocytochemical studies revealed a predominant cytoplasmic location of TWIK-1 in kidney tubular cells, neurons of vestibular ganglion in rodent cochlea and several transfected cells (Nicolas et al., 2003; Decressac et al., 2004; Nie et al., 2005). More recently TWIK-1 channels were found to localize predominantly in recycling endosomes within the cytoplasm (Feliciangeli et al., 2010). Such a distinct subcellular location was proposed to account for the lack of functional currents upon heterologous expression of TWIK-1 (Feliciangeli et al., 2010). In the present study, western blot results from two different fractionation protocols consistently showed a preferential cytoplasmic localization of TWIK-1, while only 4.82–23.59% of the channels appeared in plasma membrane fractions (Figures 8B–D). Limited by the centrifugation speed of the fractionation, small intracellular compartments (such as recycling endosome, Golgi, transport vesicles) are expected to be retained within cytosolic fractions. Thus it is reasonable to infer that some of TWIK-1 channels may well localize in one or several of these organelles in astrocytes.

Interestingly, ~40% TWIK-1 protein was detected in the mildly hydrophobic membrane proteins fraction (Figure 8C). It has been shown that the hydrophobicity of newly synthesized channel proteins changes over the course of maturation. For example, epithelial Na^+^ channel (ENaC) in the endoplasmic reticulum exists initially as a Triton X-100-soluble protein that contains high-mannose glycosylation, while it becomes a Triton X-100-insoluble protein when delivered to the cell surface (Prince and Welsh, 1999). Thus the presence of TWIK-1 in the mildly hydrophobic membrane protein fraction may reflect its trafficking between intracellular compartments and the cell surface.

In kidney, the amount of TWIK-1 in plasma membrane fractions is significantly higher than that of in hippocampus (Figures 8E,F), suggesting a variable cell surface membrane versus internal cytoplasmic pool ratio of TWIK-1 channels in different tissues. As for the mechanism transferring TWIK-1 to the cell surface, a diisoleucine repeat located in the cytoplasmic carboxyl-terminus of TWIK-1 (I293, I294) has been identified as a critical retrieval motif in both cultured kidney cells and transfected oocytes. The mutation of these sites led to greatly enhanced TWIK-1 cell surface expression and production of very large currents (Feliciangeli et al., 2010; Chatelain et al., 2012). The retrieval motif can be regulated by activation of G_*i*_-coupled receptors or interaction with EFA6, an exchange factor for the small G-protein ARF6 (Decressac et al., 2004; Feliciangeli et al., 2010). It remains to be determined whether the same signaling pathway regulates the surface expression of TWIK-1 in astrocytes.

### The molecular identity of channels underlying passive conductance remains elusive

At both of the tissue and cellular levels, we found no evidence for altered expression of the major astrocyte K^+^ channels K_ir_4.1 and TREK-1 in TWIK-1^−/−^ mice. In addition, there was no indication of up-regulation of TWIK-2 and TWIK-3, two K2P channels in the TWIK family that have the potential to compensate for TWIK-1 conductance (Figure 2). Additionally, neither K_ir_4.1 nor TREK-1 showed functional compensations in TWIK-1^−/−^mice. Under the physiological K^+^ gradient, Ba^2+^-sensitive K_ir_4.1 channels preferentially conduct inward K^+^ currents, yet 100 μM BaCl_2_ induced current inhibition and Vm depolarization did not differ between WT and TWIK-1^−/−^ mice (Figure 5). Quinine, which also inhibits TREK-1, showed comparable effect on current inhibition and Vm depolarization between WT and KO mice. Furthermore, in the absence of TWIK-1, the passive conductance remained highly permeable to Cs^+^. It should be noted that although the Cs^+^-mediated conductance showed a weak outward rectification, unlike TREK-1, this rectification does not follow GHK constant field rectification. Thus TREK-1 channels cannot be the only Cs^+^ permeable channels in TWIK-1^−/−^mice.

### Functional implication of TWIK-1 expression in astrocytes

A low level of channel gating, coupled to a predominantly cytoplasmic localization may together be responsible for the mild functional impact of TWIK-1 on astrocyte passive conductance. TWIK-1 in heterologous expression systems behaves as a classic K^+^ channel under physiological recording conditions. This contrasts with TWIK-1 in native cells, where this channel can also function as a leak Na^+^ channel. This was first revealed in kidney cells where deletion of the TWIK-1 gene produced a 6 mV hyperpolarization of the membrane potential (Millar et al., 2006). Recently a more hyperpolarized Vm was reported in pancreatic ß cells from TWIK-1^−/−^ mice (Chatelain et al., 2012). We have now shown that astrocytes in TWIK-1^−/−^ mice also have a more hyperpolarized membrane potential than those from wild type mice. This channel behavior reflects a unique attribute of TWIK-1 that has been characterized in expression systems and cultured human cardiomyocytes, where TWIK-1 can conduct Na^+^ ions in response to a fall in extracellular K^+^ concentration or in pH (Ma et al., 2011; Chatelain et al., 2012).

We found that the levels of TWIK-1 in kidney plasma membrane-containing fractions were considerably higher than in hippocampus (Figures 8E,F). This correlates with a greater hyperpolarization of the resting membrane potential on TWIK-1 knockout (−6 mV in kidney cells compared to < −1 mV in astrocytes) (Millar et al., 2006). Therefore, the amount of surface presence of TWIK-1 seemingly correlates proportionally to the degree of membrane potential depolarization in native cells.

A more negative membrane potential, compared to that of neurons, is considered essential for astrocyte homeostatic function. An important implication from this study is that TWIK-1 is unlikely to be one of those long-sought K^+^ channels subserving this function. Instead, TWIK-1 may function as a leak Na^+^ channel that counteracts the role of classic K^+^ channels in astrocytes. In view of the copious TWIK-1 expression in astrocytes, the actual function of this channel remains unknown.

Recycling of TWIK-1 between cytoplasm and cell membrane has been shown in other cell types, and the hypothesis that this serves to regulate intracellular Na^+^ content in astrocytes has emerged for future investigation. It would be interesting to know how the regulatory Na^+^ leakage via TWIK-1 affects K^+^ spatial buffering, Na^+^-dependent neurotransmitter uptake and neurovascular coupling for the critical energy metabolic regulation in the brain (Kimelberg, 2010; Pellerin and Magistretti, 2012). Our findings indicate that future work will need to identify the signaling pathways that regulate TWIK-1 trafficking, so as to reveal the physiological and pathological relevance of the TWIK-1 channel in astrocytes.

## Author contributions

Wei Wang, Adhytia Putra, Gary P. Schools and Baofeng Ma carried out the experiments. Wei Wang, Adhytia Putra and Gary P. Schools analyzed the data. Leonard K. Kaczmarek provided the TWIK-1 KO mice, discussed the project and wrote parts of the manuscript. Florian Lesage and Jacques Barhanin created the TWIK-1 KO mouse, provided consultation for genotyping, discussed the project and wrote parts of the manuscript. CH provided reagents and discussed the project. Wei Wang and Min Zhou designed the study and wrote the manuscript. Min Zhou supervised the study.

### Conflict of interest statement

The authors declare that the research was conducted in the absence of any commercial or financial relationships that could be construed as a potential conflict of interest.
